# Genetic Dissection of BDNF and TrkB Expression in Glial Cells

**DOI:** 10.3390/biom14010091

**Published:** 2024-01-11

**Authors:** Changran Niu, Xinpei Yue, Juan Ji An, Robert Bass, Haifei Xu, Baoji Xu

**Affiliations:** 1 Department of Neuroscience, The Herbert Wertheim UF Scripps Institute for Biomedical Innovation & Technology, University of Florida, Jupiter, FL 33458, USA; cniu@scripps.edu (C.N.); xyue@scripps.edu (X.Y.); anj@ufl.edu (J.J.A.); rbass@scripps.edu (R.B.); hxu3@ufl.edu (H.X.); 2 Skaggs Graduate School of Chemical and Biological Sciences, The Scripps Research Institute, Jupiter, FL 33458, USA

**Keywords:** BDNF, TrkB, Cre, glia, microglia, astrocyte, oligodendrocyte

## Abstract

The brain-derived neurotrophic factor (BDNF) and its high-affinity receptor tropomyosin-related kinase receptor B (TrkB) are widely expressed in the central nervous system. It is well documented that neurons express BDNF and full-length TrkB (TrkB.FL) as well as a lower level of truncated TrkB (TrkB.T). However, there are conflicting reports regarding the expression of BDNF and TrkB in glial cells, particularly microglia. In this study, we employed a sensitive and reliable genetic method to characterize the expression of BDNF and TrkB in glial cells in the mouse brain. We utilized three Cre mouse strains in which Cre recombinase is expressed in the same cells as BDNF, TrkB.FL, or all TrkB isoforms, and crossed them to Cre-dependent reporter mice to label BDNF- or TrkB-expressing cells with soma-localized EGFP. We performed immunohistochemistry with glial cell markers to examine the expression of BDNF and TrkB in microglia, astrocytes, and oligodendrocytes. Surprisingly, we found no BDNF- or TrkB-expressing microglia in examined CNS regions, including the somatomotor cortex, hippocampal CA1, and spinal cord. Consistent with previous studies, most astrocytes only express TrkB.T in the hippocampus of adult brains. Moreover, there are a small number of astrocytes and oligodendrocytes that express BDNF in the hippocampus, the function of which is to be determined. We also found that oligodendrocyte precursor cells, but not mature oligodendrocytes, express both TrkB.FL and TrkB.T in the hippocampus of adult mice. These results not only clarify the expression of BDNF and TrkB in glial cells but also open opportunities to investigate previously unidentified roles of BDNF and TrkB in astrocytes and oligodendrocytes.

## 1. Introduction

Brain-derived neurotrophic factor (BDNF) is a neurotrophin that is widely expressed in the central nervous system. BDNF signals via the tropomyosin-related kinase B (TrkB) receptor to regulate neuronal proliferation, differentiation and survival, and synaptic function [1]. Binding of BDNF to the full-length TrkB (TrkB.FL) receptor triggers dimerization of TrkB.FL and tyrosine autophosphorylation, leading to the activation of three main downstream signaling cascades mediated by phospholipase C γ1 (PLCγ1), phosphoinositide 3-kinase, and RAS/RAF/mitogen-activated protein kinase, respectively [2]. Alternative splicing of *Ntrk2* pre-mRNA produces the truncated TrkB (TrkB.T) isoforms that lack nearly all the intracellular domain including the tyrosine kinase motif [3]. TrkB.T can negatively regulate TrkB.FL signaling by forming a TrkB.FL-TrkB.T heterodimer and inhibiting the activation of TrkB.FL [4]. TrkB.T also functions to sequester and translocate BDNF, induce neurite outgrowth, activate downstream protein kinase C and PLCγ via G-protein, and inhibit Rho GTPase via a dissociating Rho GDP dissociation inhibitor [4]. Because of the diverse roles of the BDNF-TrkB pathway, the dysregulation of BDNF-TrkB signaling has been implicated in various disease conditions, including cognitive impairment, obesity, neurodegenerative diseases, and cancer [5].

The BDNF-TrkB signaling pathway has been studied extensively in neurons. It is well documented that neurons express BDNF, TrkB.FL, and a lower level of TrkB.T. The expression of BDNF and TrkB has also been reported in glial cells. BDNF expressed in microglia was suggested to be involved in neuropathic pain and motor-learning dependent synapse formation [6,7]. However, other studies showed very low or no expression of BDNF in microglia [8,9]. Therefore, it is debatable whether microglia express BDNF or not. It has been shown that astrocytes predominantly express TrkB.T [1,10]. Whether astrocytes also express TrkB.FL is not clear yet. Some studies have shown TrkB.FL expression in astrocytes isolated from mouse brain [11,12], whereas other studies show no TrkB.FL expression in astrocytes [13,14]. Astrocytes can internalize proBDNF secreted by neurons, convert proBDNF to mature BDNF (mBDNF), and release mBDNF for synaptic reuse [15,16]. Astrocytes were shown to express BDNF in vitro which regulates neuronal dendrite maturation [17], but whether astrocytes express BDNF in vivo under physiological conditions is unknown. Oligodendrocytes have also been shown to express BDNF, and a reduction in these impairs presynaptic vesicular exocytosis [18]. The oligodendroglial expression of TrkB is important for oligodendrocyte myelination and oligodendrocyte precursor cell (OPC) proliferation [19,20,21]. But it has not been characterized which TrkB isoform oligodendrocytes express and what percentage of oligodendrocytes express BDNF or TrkB.

To fill these knowledge gaps, in the current study, we employed a genetic method to label and characterize the expression of BDNF and TrkB in glial cells, including microglia, astrocytes, and oligodendrocytes. We crossed the *Bdnf*^2A-Cre/+^ [22], *Ntrk2*^2A-Cre/+^, and *Ntrk2*^CreER/+^ [23] mouse strains to the Cre-dependent B6;129S4-Gt(*ROSA*)*26Sor^tm9(EGFP/Rpl10a)Amc^*/J (EGFP-L10a) reporter mice [24] to generate double heterozygous mice, in which cells expressing BDNF or TrkB were labeled by EGFP. We then performed immunofluorescence staining of cell-type markers with the brain sections from these mice. The colocalization of EGFP and cell-type markers was analyzed to determine the expression of BDNF and TrkB in glial cells. There are several advantages of this method: (1) high sensitivity because a very small amount of Cre expression can induce EGFP expression in the reporter mice; (2) no involvement of BDNF and TrkB antibodies, which have non-specific immunoreactivity; (3) no need to isolate glia from the mouse brain, which involves potential contamination of other cell types; (4) being able to observe expression of BDNF and TrkB in vivo at the level of single cells.

Our results show that homeostatic and activated microglia do not express either BDNF or TrkB. Astrocytes predominantly express TrkB.T, and a small astrocyte population expresses TrkB.FL and BDNF in adult mice. A subset of oligodendrocytes express BDNF in the hippocampal CA1, as well as TrkB.T and TrkB.FL.

## 2. Materials and Methods

### 2.1. Animals

Mice of the EGFP-L10a/+ (B6;129S4-Gt(*ROSA*)*26Sor^tm9(EGFP/Rpl10a)Amc^*/J; stock #024750) and the *Bdnf*^2A-Cre/+^ (B6.FVB-*Bdnf^em1(cre)Zak^*/J; stock #030189) strains were obtained from the Jackson Laboratory. The *Ntrk2^CreER/+^* mouse strain was generously provided by Dr. David Ginty from Harvard Medical School [23]. *Ntrk2^2A-Cre/+^*, *Ntrk2^CreER/+^*, and *Bdnf^2A-Cre/+^* mice were crossed to EGFP-L10a/+ mice to generate *Ntrk2^2A-Cre/+^*;EGFP-L10a/+, *Ntrk2^CreER/+^*;EGFP-L10a/+, and *Bdnf^2A-Cre/+^*;EGFPL10a/+ mice. Both male and female mice that were 2–6 months old were used in the experiments.

### 2.2. Generation of Ntrk2^2A-Cre/+^ Mice 

We generated a *Ntrk2^2A-Cre^* mouse allele in which the DNA sequence encoding P2A-Cre recombinase was inserted immediately before the stop codon for TrkB.FL at the *Ntrk2* locus using the CRISPR/Cas9 technique. sgRNA (5′-AGTCAAGAGGTTCGTCGTGT-3′) was designed using the CRISPR tool (http://crispr.mit.edu) to minimize potential off-target effects. The DNA donor plasmid contains two homology arms with 840 bp flanking the P2A-Cre sequence, which were cloned into BamHI-digested pBluescript II KS (-) vector using the Gibson assembly method (NEB, Ipswich, MA, USA, E5510). To block further Cas9 targeting and recutting after undergoing homology-direct repair, we introduced a silent mutation into the PAM motif of the sgRNA located within the 3′ homology arm in the donor plasmid by using Q5 Site-Directed Mutagenesis kit (NEB, E0054). The donor plasmid was confirmed with DNA sequencing. Microinjection of a mixture of sgRNA, donor DNA, and Cas9 protein was performed by the Genomic Modification Facility at Scripps Research. Zygotes were cultured to the blastocyst stage in vitro. Genomic DNA from blastocysts was extracted, and PCR screen was performed to select blastocysts with successful homologous recombination. Positive blastocysts were transferred into the oviduct of pseudo-pregnant females to produce founder mice. Two out of twenty-eight pups were positive for the knockin, confirmed by the genomic DNA PCR using the following primers: CCTCCTGGTGAGCAAACGAT (forward) and GACATAGGGCCGGGATTCTC (reverse) for PCR across the 5′ homology arm, and CACCTCCATGCCTGTGTTTT (forward) and GGTTCTTGCGAACCTCATCA (reverse) for PCR across the 3′ homology arm. The founder mice were crossed to C57BL/6J to produce mice with germline transmission. Cre expression in these lines was confirmed by in situ hybridization.

### 2.3. In Situ Hybridization

Freshly dissected mouse brains were quickly frozen in a 2-methylbutane (Fisher Scientific, Hampton, VA, USA, #O3551-4) dry ice bath. Subsequently, 14 μm thick cryostat sections were collected onto Superfrost Plus slides (Fisherbrand, Waltham, MA, USA, #12-550-15) and used for in situ hybridization. Fluorescence in situ hybridization was performed using the RNAscope Multiplex Fluorescent Reagent Kit v2 (#323100, Advanced Cell diagnostics, Newark, CA, USA). Brain sections were fixed with pre-chilled 10% formalin at 4 °C for 15 min and dehydrated with 50%, 75%, and 100% ethanol. Sections were air-dried for 20 min and followed by v2Hydrogen peroxide for 10 min and pretreated with protease IV for 30 min, washed 2 times in PBS, and incubated with the target probes (Ntrk2-C3, #423611-C3; Cre-C2, #31228-C2) for 2 h at 40 °C. Signals were amplified by subsequent incubation with v2Amp1 for 30 min, v2Amp2 for 30 min, v2Amp3 for 15 min, v2C2/C3HPR for 15 min, followed by TSA Plus Fluorescein/Cyanine5 (NEL754001KT, Akoya Biosciences, Marlborough, MA, USA) for 30 min at 40 °C. Slides were counterstained with DAPI, and images were acquired using a Nikon C2+ confocal microscope.

### 2.4. Immunohistochemistry

Mice were perfused with phosphate-buffered saline (PBS) and then 4% paraformaldehyde (PFA) in PBS. The brain was dissected and postfixed overnight in 4% PFA-PBS. Mouse brains were soaked in 30% sucrose-PBS for cryoprotection. Brains were sectioned into 40 μm thick slices using a sliding microtome (Leica). Brain sections were blocked in 0.3% Triton X-100 and 10% normal horse serum before overnight incubation in primary antibody at 4 °C. The following primary antibodies were used: guinea pig anti-Iba1 (1:500; Synaptic System, Göttingen, Germany, #234308), goat anti-SOX9 (1:2000; R&D Systems, Minneapolis, MN, USA, #AF3075), mouse anti-GFAP (1:500; Cell Signaling Technology, Danvers, MA, USA, #3670S), mouse anti-Olig2 (1:250; Millipore, Burlington, MA, USA, #MABN50), rabbit anti-ASPA (1:1500; GeneTex, Irvine, CA, USA, #GTX113389), chicken anti-GFP (1:2000; abcam, Cambridge, UK, #13970), and rabbit anti-DsRed (1:1000; TaKaRa, San Jose, CA, USA, #632496). The brain sections were washed three times with PBS before incubation in secondary antibody for 1 h at room temperature. The brain sections were then counterstained with DAPI, mounted onto microscope slides, and covered with mounting media. 

### 2.5. LPS Injection

LPS (Millipore #L6529) was dissolved in sterile saline to a final concentration of 0.1 μg/μL. The mice at 2–6 months old were divided into two sex- and age-matched groups and given daily injection of saline or LPS at a dose of 500 μg/kg body weight for seven consecutive days. The mice were harvested the next day after the last treatment. 

### 2.6. Tamoxifen Injection

Tamoxifen (Sigma, St. Louis, MO, USA, #T5648-5G) was dissolved in 100% ethanol to a concentration of 20 mg/mL. An equal volume of corn oil was added to the tamoxifen solution. The corn oil/ethanol mixture was vortexed, centrifuged for 10 min at 13,000 rpm, and then placed in a vacuum centrifuge for 30 min to evaporate ethanol. Tamoxifen was given to *Ntrk2^CreER/+^*;EGFP-L10a/+ mice at the age of 6 weeks through daily i.p. injection at a dose of 150 mg/kg body weight for 10 consecutive days to induce translocation of the Cre-ER fusion protein to the nucleus. The mice were given three weeks to recover before they were harvested. 

### 2.7. Plasmids and Viruses

pAAV8-EF1a-Nuc-flox(mCherry)-EGFP was obtained from Addgene, Watertown, MA, USA (plasmid #112677, a gift from Brandon Harvey [25]). For higher expression in glial cells, the EF1a promoter was replaced by the CAG promoter from pAAV-CAG-mNeonGreen (Addgene plasmid #99134, a gift from Viviana Gradinaru [26]). pAAV8-EF1a-Nuc-flox(mCherry)-EGFP was digested with AgeI and BamHI, and the CAG promoter fragment was amplified by PCR (forward primer: GAATACCGGTCGTTACATAACTTACGGTAAATG; reverse primer: GAATGGATCCCGCCCGCCGCGCGCTT). PCR product was digested with AgeI and BamHI and ligated into the backbone of pAAV8-EF1a-Nuc-flox(mCherry)-EGFP. The resulting pAAV8-CAG-Nuc-flox(mCherry)-EGFP plasmid was confirmed by sequencing and used to package AAV viruses. AAV viruses were purified with TaKaRa AAVpro Purification Kit Maxi (#6666).

### 2.8. Stereotaxic Injection of Adeno-Associated Virus

Mice were anesthetized using isoflurane and securely positioned on a stereotaxic holder (David Kopf, Tujunga, CA, USA, Model 940). A small incision was made to expose the skull, and subsequently, a small hole was drilled on the skull above an injection site. For injection, a Nanofil 33-gauge needle (World Precision Instruments, Sarasota, FL, USA, #NF33BV-2) was slowly inserted into the CA1 region (AP: −1.60 mm; ML: ±0.80 mm; DV: −1.80 mm). AAV8-CAG-Nuc-flox(mCherry)-EGFP (150 nL at 3.12 × 10^12^ vg/mL) was then injected at a rate of 30 nL/min using a micro syringe pump (World Precision Instruments, SYS-MICRO4). The needle was then withdrawn after staying in the same position for 5 min. Following the injection, mice received Loxicom (5 mg/kg) for analgesia and were returned to their home cages.

### 2.9. Imaging and Cell Number Quantification

Fluorescent images were captured using a Nikon C2+ confocal microscope. CD68 intensity was quantified in FIJI software (version 2.1.0/1.53q) and normalized to average CD68 intensity of microglia in saline control mice. Cell numbers were quantified using the cell counter plugin in FIJI software. 

### 2.10. Statistical Analyses

Statistical analyses were performed using Prism 10 for macOS. Unpaired *t*-test was used to compare relative CD68 intensity per microglia in saline control mice and in LPS-injected mice. The minimal level of significance was set at *p* < 0.05.

## 3. Results

### 3.1. Homeostatic and Activated Microglia in the Cortex, Hippocampus, and Spinal Cord Do Not Express BDNF 

**Figure 1:**
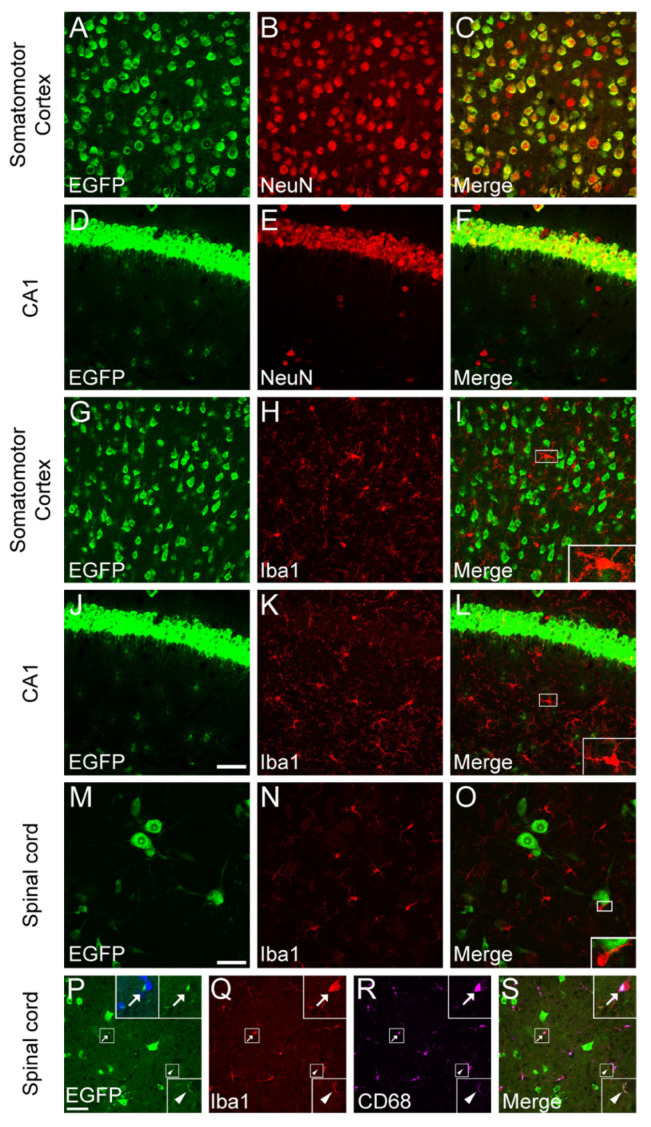
Homeostatic microglia do not express BDNF. (**A**–**F**) BDNF expression in NeuN^+^ neurons in the somatomotor cortex (MO) (**A**–**C**) and CA1 (**D**–**F**). (**G**–**O**) Homeostatic microglia do not express BDNF in the MO (**G**–**I**), CA1 (**J**–**L**), and spinal cord (**M**–**O**). Inserts in (**I**,**L**,**O**) are enlarged 3×. (**P**–**S**) Colocalization of EGFP puncta signals with CD68^+^ microglia lysosomes. Arrows denote EGFP signal inside microglial cytoplasm. Arrowheads denote EGFP signal inside microglial processes. Upper inserts are enlarged 3×, and lower inserts are enlarged 2.5×. Scale bar = 50 μm. n = 4 mice.

*Bdnf^2A-Cre/+^* mice that express Cre recombinase in BDNF-expressing cells were crossed to Cre-dependent EGFP-L10a/+ reporter mice to generate *Bdnf^2A-Cre/+^*;EGFP-L10a/+ mice. Cre-mediated excision of *loxP*-flanked STOP sequence results in constitutive expression of the EGFP-L10a fusion protein localized in the nucleus and cytoplasm of Cre-expressing cells. EGFP is, thus, expressed in cells that express BDNF at any time during the lifespan of a *Bdnf^2A-Cre/+^*;EGFP-L10a/+ mouse. The localization of EGFP in cells bodies facilitates visualization of the co-localization between EGFP and cell-type markers. We first used NeuN as a marker to label neurons. As expected, most neurons in the cortex and hippocampal CA1 express BDNF (Figure 1A–F), validating the accuracy of this approach for the examination of BDNF expression. 

Next, we wanted to know whether microglia express BDNF. For this, we used Iba1 antibody to label microglia in *Bdnf^2A-Cre/+^*;EGFP-L10a/+ mice. We examined BDNF expression in the somatomotor cortex (MO), hippocampal CA1, and spinal cord because microglia in these regions have been suggested to express BDNF [6,7]. Surprisingly, we did not observe EGFP expression in the nuclei of Iba1^+^ microglia in the MO, hippocampal CA1, or the spinal cord of 4-month-old *Bdnf^2A-Cre/+^*;EGFP-L10a/+ mice, indicating that homeostatic microglia do not express BDNF under physiological conditions (Figure 1G–O). However, we did observe EGFP puncta in some microglial cell bodies and processes, outside microglial nuclei (Figure 1P–S). Immunostaining of CD68, a microglial lysosomal marker, showed that the EGFP puncta are always colocalized with CD68 (Figure 1P–S). This indicates that these EGFP signals in microglia come from BDNF-expressing cells engulfed by microglia and are not from BDNF expression in microglia. 

**Figure 2:**
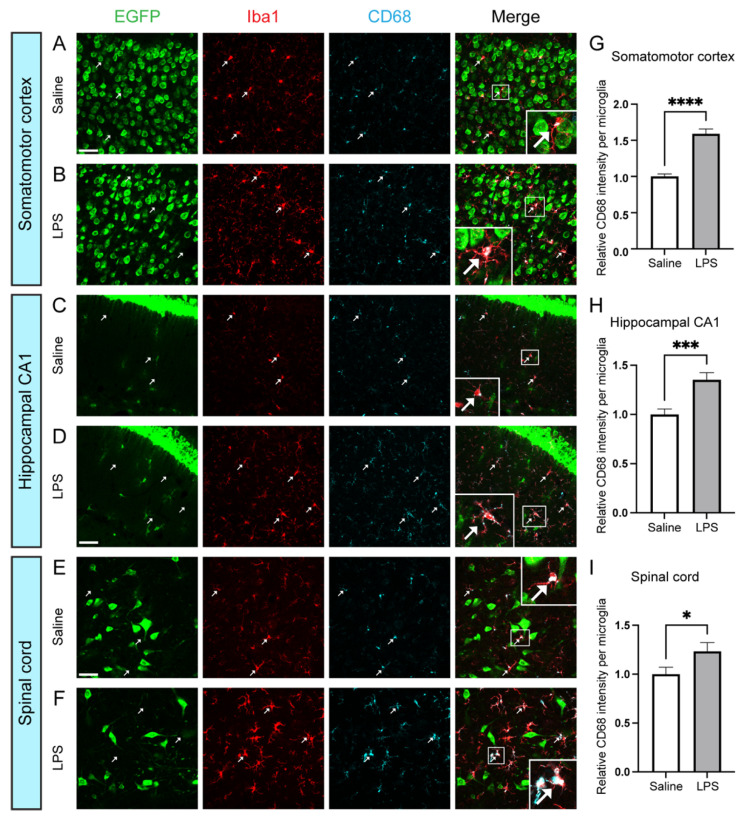
Activated microglia do not express BDNF. (**A**–**F**) Immunostaining of Iba1 and CD68 in the somatomotor cortex (**A**,**B**), hippocampal CA1 (**C**,**D**), and spinal cord (**E**,**F**) of *Bdnf^2A-Cre/+^*;EGFP-L10a/+ mice injected with saline (**A**,**C**,**E**) or LPS (**B**,**D**,**F**). Arrows indicate colocalization of Iba1 and CD68, and no EGFP inside microglia. Inserts in (**A**,**B**,**E**,**F**) are enlarged 3× and inserts in (**C**,**D**) are enlarged 2.5×. Scale bar = 50 μm. (**G**–**I**) Quantification of relative CD68 intensity per microglia. n = 3 mice for both saline and LPS treatments. **** *p* < 0.0001, *** *p* = 0.0001, and * *p* = 0.0439 by two-sided *t*-test. Data are shown as mean ± SEM.

Microglia can exist in homeostatic or activated states. To investigate whether activated microglia express BDNF under pathological conditions, we injected lipopolysaccharide (LPS) to induce microglial activation in adult *Bdnf^2A-Cre/+^*;EGFP-L10a/+ mice. CD68 is used as a marker for microglia activation, as activated microglia express a higher level of CD68. LPS treatment resulted in a significant increase in CD68 intensity per microglia (Figure 2). We did not observe EGFP signals in Iba1^+^ microglial nuclei in LPS-injected mice, suggesting that activated microglia also do not express BDNF (Figure 2B,D,F). Therefore, our results suggest that both homeostatic and activated microglia do not express BDNF.

### 3.2. Some Astrocytes Express BDNF in the Cortex and Hippocampus

**Figure 3:**
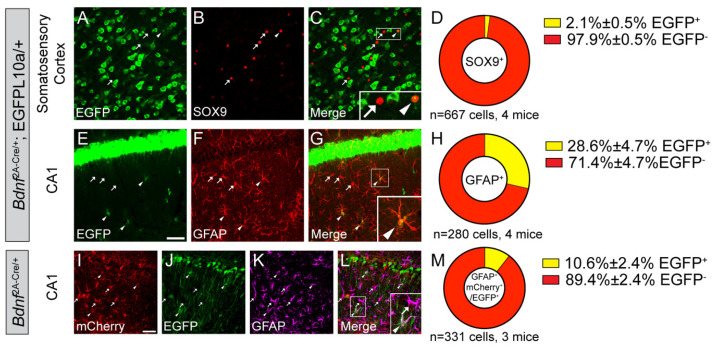
A small subset of astrocytes express BDNF in the hippocampus. (**A**–**H**) Immunostaining of SOX9 (**A**–**C**) and GFAP (**E**–**G**) in the SS (**A**–**C**) and CA1 (**E**–**G**) of *Bdnf^2A-Cre/+^*;EGFP-L10a/+ mice, and (**D**,**H**) quantification of colocalization (mean ± SEM). Inserts in (**C**,**G**) are enlarged 2.5×. (**I**–**M**) Immunostaining of GFAP in the CA1 of adult *Bdnf^2A-Cre/+^* mice injected with the mCherry-to-EGFP color switch AAV, and (**M**) quantification of colocalization (mean ± SEM). Insert in (**L**) is enlarged 2×. Scale bars = 50 μm. Arrowheads denote EGFP-expressing astrocytes. Arrows denote astrocytes that do not express EGFP.

Whether astrocytes express BDNF in vivo under physiological conditions is unknown. To answer this question, we labeled astrocytes in *Bdnf^2A-Cre/+^*;EGFP-L10a/+ mice using SOX9 antibody for the somatosensory cortex (SS) and GFAP antibody for the hippocampus. As GFAP expression in the cortex is low and does not mark astrocytes well, SOX9 was used for an astrocyte marker in the cortex. Although SOX9 is expressed in neural stem cells, it is expressed exclusively in astrocytes outside of the adult neurogenic regions [27]. We observed that a small portion of astrocytes (2.1% in the SS and 28.6% in the CA1) are EGFP^+^ in the *Bdnf^2A-Cre/+^*;EGFP-L10a/+ mice, indicating that these cells express BDNF at some point during the lifetime of the mice (Figure 3A–H). The EGFP signals in these mice are cumulative, because once the EGFP expression in a cell is turned on by Cre, it is never turned off, even if the cell stops to express Cre. Therefore, we cannot know when BDNF is expressed in these mice.

To test whether astrocytes in adult mice still express BDNF, we injected a Cre-dependent mCherry-to-EGFP color switch adeno-associated virus (AAV), AAV8-CAG-Nuc-flox(mCherry)-EGFP, into the hippocampal CA1 of 2-month-old *Bdnf^2A-Cre/+^* mice. In these mice, transduced Cre-negative cells will express mCherry, while transduced Cre-positive cells will express EGFP. EGFP expressed from the virus is not restricted by L10a protein and, thus, labels both cell bodies and neuronal processes. We chose the CA1 rather than the SS to examine adult astrocytic BDNF expression because there were more EGFP-labeled astrocytes in the CA1 than in the SS of *Bdnf^2A-Cre/+^*;EGFP-L10a/+ mice (Figure 3A–H). Approximately 87% astrocytes were transduced by the AAV, and 10.6% of the transduced astrocytes (GFAP^+^ and mCherry^+^/EGFP^+^) express EGFP in adult mice (Figure 3I–M). This suggests that a small portion of astrocytes in the CA1 still express BDNF in adult mice. Because this number is smaller than the cumulative 28.6% in *Bdnf^2A-Cre/+^*;EGFP-L10a/+ mice, there could be two non-exclusive possibilities: (1) some astrocytes express BDNF during development and stop BDNF expression in adulthood; (2) there are several small subsets of mature astrocytes that transiently express BDNF at different times. To figure out which possibility is true, future studies could investigate BDNF expression during the development. 

### 3.3. A Small Portion of Oligodendrocytes Express BDNF in the Hippocampal CA1 Subregion 

**Figure 4:**
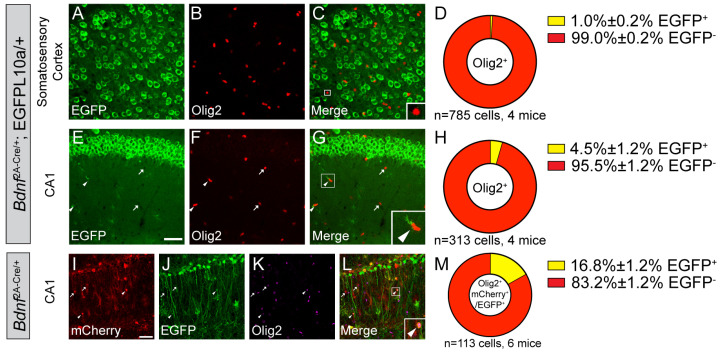
A small subset of oligodendrocytes express BDNF in the hippocampus. (**A**–**H**) Immunostaining of Olig2 in the SS (**A**–**C**) and CA1 (**E**–**G**) of adult *Bdnf^2A-Cre/+^*;EGFP-L10a/+ mice, and (**D**,**H**) quantification of colocalization (mean ± SEM). (**I**–**M**) Immunostaining of Olig2 in the CA1 of adult *Bdnf^2A-Cre/+^* mice injected with the mCherry-to-EGFP color switch AAV, and (**M**) quantification of colocalization (mean ± SEM). Insert in (**C**) is enlarged 3×, and inserts in (**G**,**L**) are enlarged 2.5×. Scale bars = 50 μm. Arrowheads denote EGFP-expressing oligodendrocytes. Arrows denote oligodendrocytes that do not express EGFP.

Next, we examined BDNF expression in Olig2^+^ oligodendrocytes. We found that most Olig2^+^ oligodendrocytes in the SS do not express BDNF, and 4.5% of Olig2^+^ oligodendrocytes in the CA1 express BDNF at some point during the lifespan of *Bdnf^2A-Cre/+^*;EGFP-L10a/+ mice (Figure 4A–H). To find out whether these oligodendrocytes express BDNF in adult mice, we injected the mCherry-to-EGFP color switch virus to the hippocampus of *Bdnf^2A-Cre/+^* mice and examined colocalization of Olig2 with mCherry and EGFP. Out of the transduced Olig2^+^ cells, 16.8% express BDNF in adult mice (Figure 4I–M). It is unexpected that the percentage of BDNF-expressing oligodendrocytes in adults is higher than that of cumulative expression in *Bdnf^2A-Cre/+^*;EGFP-L10a/+ mice. One possible explanation is that AAV injection might cause axonal damage, which induces myelination and the generation of BDNF-expressing oligodendrocytes. 

### 3.4. Generation of Ntrk2^2A-Cre^ Allele

**Figure 5:**
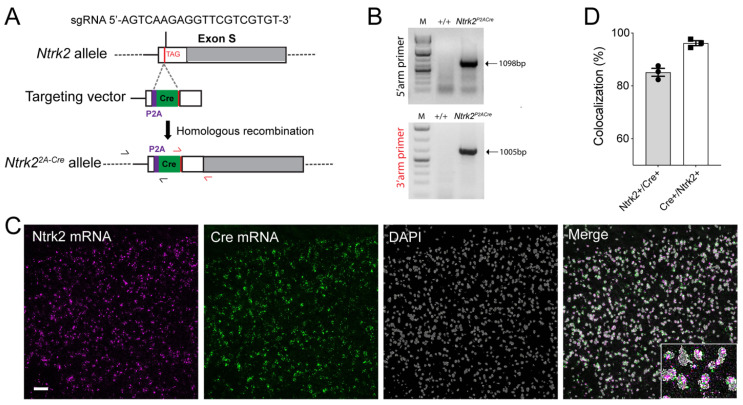
Generation of *Ntrk2^2A-Cre^* mice. (**A**) Strategy for insertion of the P2A-Cre sequence into the *Ntrk2* locus. (**B**) Genomic DNA PCR to identify *Ntrk2^2A-Cre/+^* mice. One primer is located outside each of the two homology arms in the targeting vector. (**C**,**D**) In situ hybridization of the cortex. The insert in (**C**) is enlarged 4×. Scale bar = 50 μm.

To label cells that express TrkB.FL, we generated the *Ntrk2^2A-Cre/+^* mouse strain, which expresses Cre recombinase in TrkB.FL-expressing cells. We employed the CRISPR/Cas9 technology to insert the P2A-Cre sequence into the *Ntrk2* locus immediately before the stop codon for the TrkB.FL (Figure 5A). Mice with the insertion were identified using genomic DNA PCR (Figure 5B). There was a high colocalization between *Cre* mRNA and *Ntrk2* mRNA for TrkB-FL in the cortex of *Ntrk2^2A-Cre/+^* mice (Figure 5C,D), validating the allele.

### 3.5. Microglia in the Cortex, Hippocampus, or Spinal Cord Do Not Express TrkB.FL or TrkB.T

**Figure 6:**
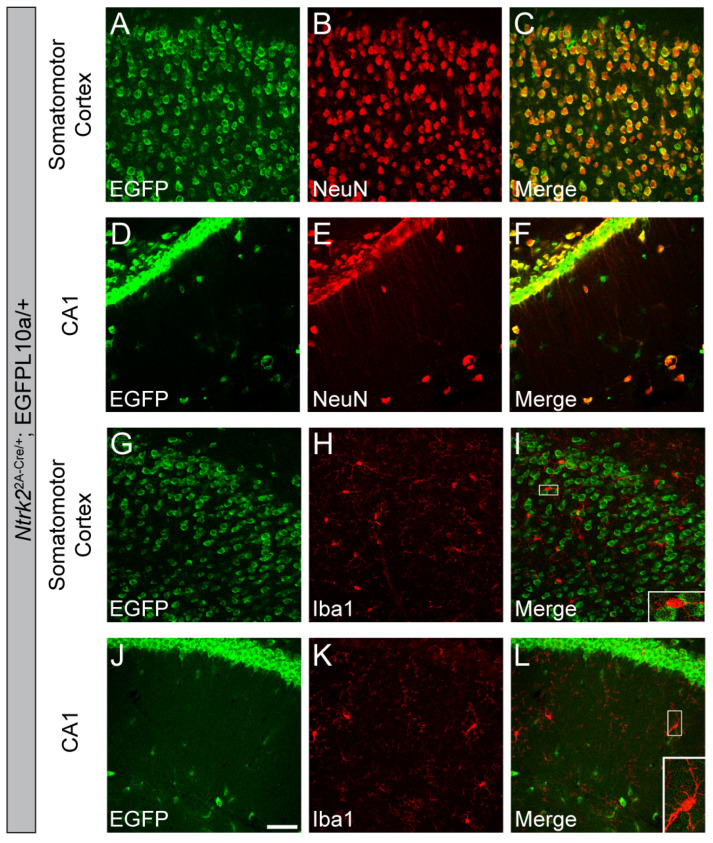
Microglia do not express TrkB.FL. (**A**–**F**) Colocalization of NeuN^+^ neurons and EGFP in the MO and CA1 of *Ntrk2^2A-Cre/+^*;EGFP-L10a/+ mice. (**G**–**L**) Homeostatic microglia do not express EGFP in the MO and CA1 of *Ntrk2^2A-Cre/+^*;EGFP-L10a/+ mice. Inserts in (**I**,**L**) are enlarged 3×. Scale bar = 50 μm. n = 4 mice.

To study TrkB.FL expression in glial cells, we crossed *Ntrk2^2A-Cre/+^* mice that express Cre in TrkB.FL-expressing cells to the Cre-dependent EGFP-L10a/+ reporter mice to generate *Ntrk2^2A-Cre/+^*;EGFP-L10a/+ mice in which TrkB.FL expression is genetically labeled by EGFP. As expected, all neurons in the MO and hippocampal CA1 express TrkB.FL (Figure 6A–F). Next, we examined whether microglia express TrkB.FL. No colocalization of EGFP and Iba1 was found in the MO or hippocampal CA1 of 4-month-old *Ntrk2^2A-Cre/+^*;EGFP-L10a/+ mice, indicating that microglia in these regions do not express TrkB.FL any time up to the time when the mice are perfused (Figure 6G–L). 

**Figure 7:**
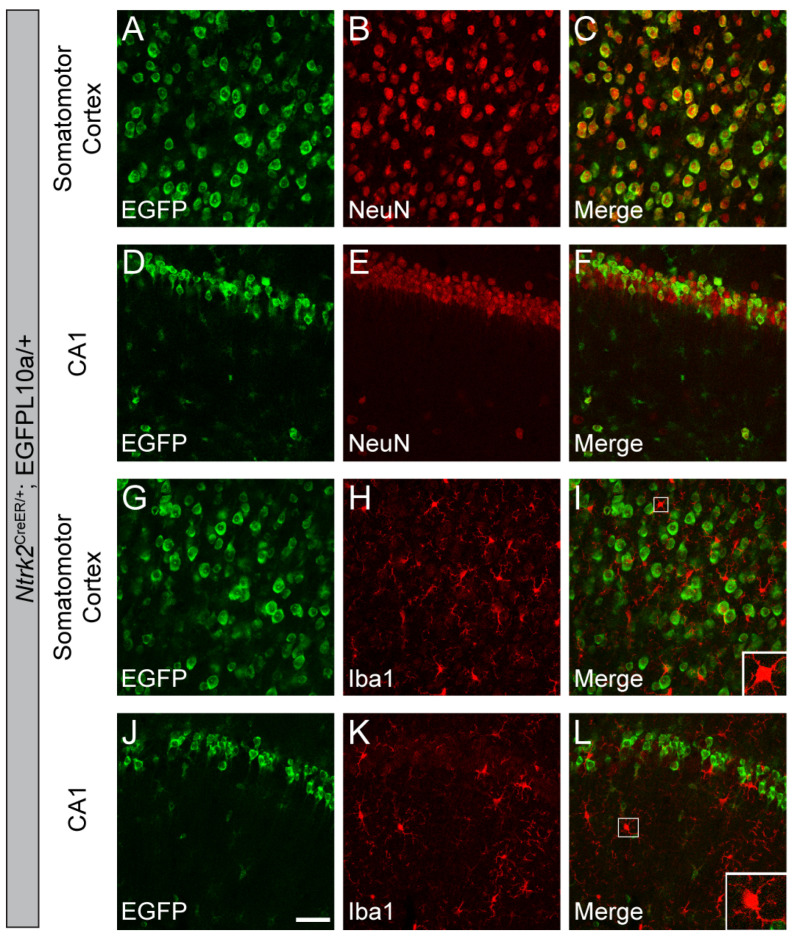
Microglia do not express either TrkB.FL or TrkB.T in adult mice. (**A**–**F**) Colocalization of NeuN^+^ neurons and EGFP in the MO and CA1 of *Ntrk2^CreER/+^*;EGFP-L10a/+ mice. (**G**–**L**) Homeostatic microglia are not labeled by EGFP in the MO and CA1 of *Ntrk2^CreER/+^*;EGFP-L10a/+ mice. Inserts in (**I**,**L**) are enlarged 3×. Scale bar = 50 μm. n = 4 mice.

To see whether microglia express TrkB.T, we utilized *Ntrk2^CreER/+^* mice that express Cre-ER under the *Ntrk2* promoter. We crossed *Ntrk2^CreER/+^* mice to Cre-dependent EGFP-L10a/+ reporter mice to generate *Ntrk2^CreER/+^*;EGFP-L10a/+ mice, in which cells expressing either TrkB.FL or TrkB.T are genetically labeled by EGFP upon tamoxifen exposure. As expected, neurons in the cortex and hippocampal CA1 are labeled by EGFP due to their expression of TrkB.FL and TrkB.T. However, not all neurons were labeled by EGFP (Figure 7A–F). Because all neurons in the MO and CA1 express TrkB.FL in adult mice (Figure 6A–F), this suggests that the Cre induction by tamoxifen was lower than 100%. Our data showed no colocalization of EGFP and Iba1 in either the MO or CA1 regions (Figure 7G–L). This means that Iba1^+^ microglia do not express either TrkB.FL or TrkB.T in adult mice. 

### 3.6. Astrocytes Predominantly Express TrkB.T and a Small Subset of Astrocytes Express TrkB.FL 

**Figure 8:**
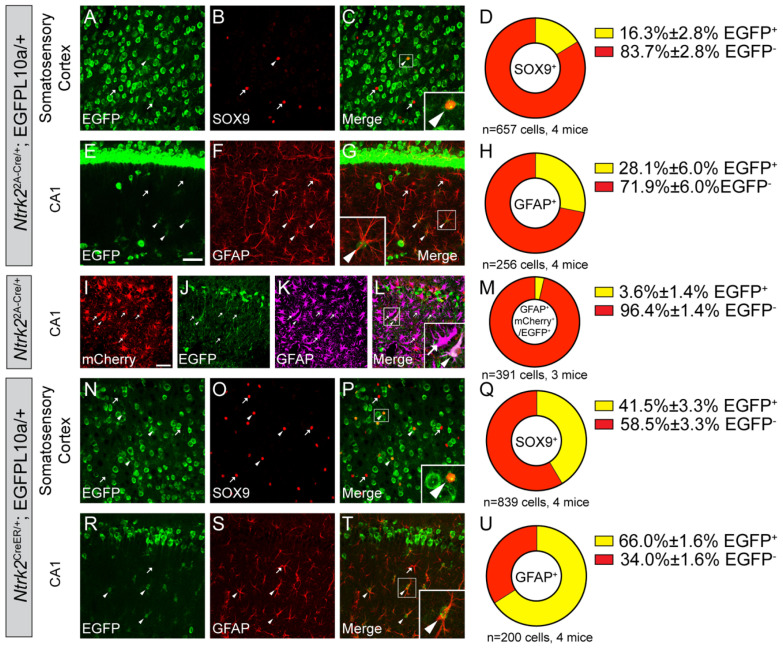
Astrocytes predominantly express TrkB.T in adult mice. (**A**–**G**) Immunostaining of SOX9 (**A**–**C**) or GFAP (**D**–**G**) in the SS (**A**–**C**) and CA1 (**E**–**G**) of *Ntrk2^2A-Cre/+^*;EGFP-L10a/+ mice, and (**D**,**H**) quantification of colocalization (mean ± SEM). (**I**–**M**) Immunostaining of GFAP in the CA1 of adult *Ntrk2^2A-Cre/+^* mice injected with the mCherry-to-EGFP color switch AAV, and the quantification of the colocalization of GFAP and EGFP (mean ± SEM). (**N**–**U**) Immunostaining of SOX9 (**A**–**C**) or GFAP (**D**–**G**) in the SS (**A**–**C**) and CA1 (**E**–**G**) of *Ntrk2^CreER/+^*;EGFP-L10a/+ mice, and the quantification of colocalization (mean ± SEM). Inserts in (**C**,**P**) are enlarged 3×, and inserts in (**G**,**L**,**T**) are enlarged 2.5×. Scale bars = 50 μm. Arrowheads denote EGFP-expressing astrocytes. Arrows denote astrocytes that do not express EGFP.

Astrocytes have been shown to predominantly express TrkB.T; however, whether astrocytes also express TrkB.FL is not as clear. To investigate the expression of TrkB.FL in astrocytes, we quantified the colocalization of EGFP and astrocyte markers, SOX9 and GFAP, in *Ntrk2^2A-Cre/+^*;EGFP-L10a/+ mice. Our analyses showed that a small portion of astrocytes (16.3% in SS and 28.1% in hippocampal CA1) express TrkB.FL at some point during the lifespan of the mice (Figure 8A–H). To test whether astrocytes in adult mice express TrkB.FL, we injected the Cre-dependent mCherry-to-EGFP color switch AAV to the hippocampus of 2-month-old *Ntrk2^2A-Cre/+^* mice [25]. Further, 92.7% of astrocytes were transduced by the AAV virus, and only 3.6% of the transduced cells expressed Cre (Figure 8I–M). These results suggest that only a very small subset of astrocytes express TrkB.FL in adult mice. Because this number is smaller than the 18.0% seen in *Ntrk2^2A-Cre/+^*;EGFP-L10a/+ mice, the two non-exclusive possibilities discussed before may also apply here: (1) some astrocytes express TrkB.FL during development and stop the expression in adulthood; (2) there are several small subsets of mature astrocytes that transiently express TrkB.FL at different times.

Next, we examined the expression of TrkB.T using *Ntrk2^CreER/+^*;EGFP-L10a/+ mice where cells expressing either TrkB.FL or TrkB.T were labeled by EGFP. Tamoxifen was given at the age of 6 weeks to induce Cre recombination. We found that 41.5% astrocytes express either TrkB.FL or TrkB.T in the SS and 66.0% in the CA1 (Figure 8N–U). Because we showed that most astrocytes do not express TrkB.FL in the CA1 of adult mice (Figure 8I–M), this means most of the EGFP^+^ astrocytes in the *Ntrk2^CreER/+^*;EGFP-L10a/+ mice express TrkB.T only. Due to the incomplete Cre induction described above, the actual percentage of astrocytes that express TrkB in *Ntrk2^CreER/+^*;EGFP-L10a/+ mice could be higher than what we show. 

### 3.7. Oligodendrocytes Express TrkB.FL and TrkB.T in Adult Brains 

**Figure 9:**
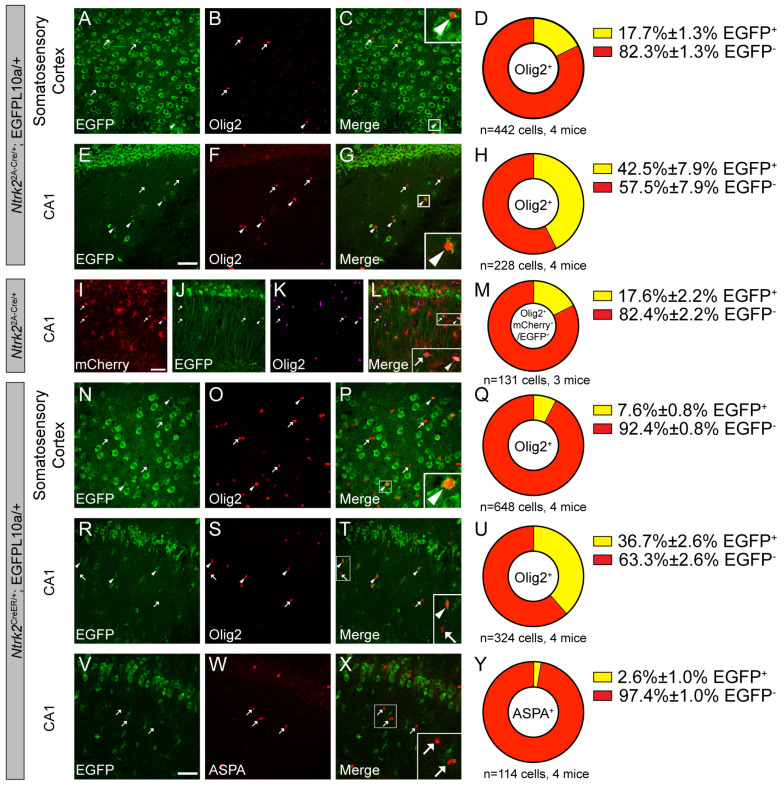
OPCs express TrkB.FL and TrkB.T in adult mice. (**A**–**G**) Immunostaining of Olig2 in the SS (**A**–**C**) and CA1 (**E**–**G**) of *Ntrk2^2A-Cre/+^*;EGFP-L10a/+ mice, and (**D**,**H**) quantification of colocalization (mean ± SEM). (**I**–**M**) Immunostaining of Olig2 in the CA1 of adult *Ntrk2^2A-Cre/+^* mice injected with the mCherry-to-EGFP color switch AAV, and quantification of the colocalization of GFAP and EGFP (mean ± SEM). (**N**–**U**) Immunostaining of Olig2 in the SS (**N**–**P**) and CA1 (**R**–**T**) of *Ntrk2^CreER/+^*;EGFP-L10a/+ mice, and (**Q**,**U**) quantification of colocalization (mean ± SEM). (**V**–**Y**) Immunostaining of ASPA in the CA1 (**V**–**X**) of *Ntrk2^CreER/+^*;EGFP-L10a/+ mice, and (**Y**) quantification of colocalization. Inserts in (**C**,**G**,**P**) are enlarged 3×, and inserts in (**L**,**T**,**X**) are enlarged 2×. Scale bars = 50 μm. Arrowheads denote EGFP-expressing oligodendrocytes. Arrows denote oligodendrocytes that do not express EGFP.

Lastly, we investigated TrkB expression in oligodendrocytes. The colocalization of the oligodendrocyte marker Olig2 and EGFP in *Ntrk2^2A-Cre/+^*;EGFP-L10a/+ mice suggests that a subset of oligodendrocytes (17.7% in SS and 42.5% in CA1) express TrkB.FL at some point during their lifespan (Figure 9A–H). To examine whether oligodendrocytes express TrkB.FL in adult mice, the Cre-dependent mCherry-to-EGFP color switch AAV was injected into the hippocampus of adult *Ntrk2^2A-Cre/+^* mice. The transduction rate of oligodendrocytes is lower than that of astrocytes. Around 25.7% of oligodendrocytes were transduced by the AAV. We observed that 17.6% of transduced Olig2^+^ oligodendrocytes expressed TrkB.FL in the adult mouse brains (Figure 9I–M). 

To find out whether oligodendrocytes also express TrkB.T, we quantified colocalization of the Olig2 and EGFP in tamoxifen-treated *Ntrk2^CreER/+^*;EGFP-L10/+ mice. We found that 7.6% of Olig2^+^ oligodendrocytes are colocalized with EGFP in the SS (Figure 9N–Q), indicating that these cells express either TrkB.FL or TrkB.T in the adult mice. In CA1, 36.7% of Olig2^+^ oligodendrocytes express either TrkB.FL or TrkB.T in the adult mice (Figure 9R–U). Because we showed 17.6% of Olig2^+^ oligodendrocytes express TrkB.FL in the CA1 of adult mice, this means that there are at least another 21% of Olig2^+^ oligodendrocytes that express only TrkB.T in the CA1 of adult mice. Olig2 labels both oligodendrocyte precursor cells (OPCs) and mature oligodendrocytes. To differentiate between these two populations, we used aspartoacylase (ASPA) as a marker to mark mature oligodendrocytes. Our results show that nearly all mature oligodendrocytes do not express either TrkB.FL or TrkB.T in the CA1 of adult mice, which suggests that only OPCs express TrkB.FL and TrkB.T in adult mice (Figure 9V–Y). This finding also explains why more oligodendrocytes in the CA1 were labeled by EGFP in *Ntrk2^2A-Cre/+^*;EGFP-L10a/+ mice than in AAV-injected *Ntrk2^2A-Cre/+^* mice (Figure 9E–M). 

## 4. Discussion

Whether microglia express BDNF and TrkB or not is controversial. Microglia were reported to express BDNF in vitro at the level of mRNA [28,29] and protein [30] decades ago. LPS stimulation was suggested to increase BDNF secretion from cultured microglia [28,30]. In vivo experiments showed BDNF expression in activated microglia [31]. BDNF from spinal microglia was suggested to be responsible for pain hypersensitivity [7]. Microglial BDNF was also reported to promote motor learning-dependent synapse formation [6]. Following these studies, many researchers showed that the knockdown of microglial expression of BDNF disrupts biological processes, including the self-renewal and proliferation of hippocampal neurons [32], nerve injury-induced pyramidal neuron hypersensitivity in the SS cortex [33], and mechanical allodynia induced by high-frequency stimulation [34]. Microglial BDNF was also suggested to be critical for (*R*)-ketamine-mediated microglial activation in the medial prefrontal cortex of chronic social defeat stress mice [35]. However, other studies using *Bdnf^Laz/+^* reporter mice and transcriptomic analysis show that microglia do not express significant levels of BDNF in the spinal cord [8,36]. This discrepancy likely comes from the presence of non-specific immunoreactivity of BDNF antibodies, as many previous efforts to characterize BDNF expression relied on immunohistochemistry. To avoid the use of BDNF antibodies and increase detection sensitivity, we took advantage of the *Bdnf^2A-cre/+^* knockin mice that express Cre recombinase under the *Bdnf* promoter and the EGFP-L10a/+ reporter mice that express EGFP localized in cell bodies but not dendrites and axons for better visualization of colocalization. Our data suggest that both resting and activated microglia do not express BDNF in the examined CNS regions, including the MO cortex, hippocampal CA1, and spinal cord. These results are consistent with findings from transcriptomic analysis that showed microglia do not express BDNF in the spinal cord [8], and from another group that recently demonstrated resting microglia and microglia activated by ATP do not express BDNF transcriptionally and translationally in the mouse motor cortex [9]. Therefore, the phenotypes seen after deleting the microglial *Bdnf* gene likely come from other effects. However, our data do not exclude the possibility that microglia may have the potential to internalize and recycle BDNF expressed by neurons and other cell types in the CNS. 

Using the same approach, we also show that microglia do not express the TrkB receptor in the CNS regions we examined. Our data show microglia do not express TrkB.FL from embryo to adult and do not express TrkB.T in adult mice. This means that at least in adult mice, microglia do not respond to BDNF through the TrkB receptor. However, it is still likely that BDNF can signal through the low-affinity receptor p75^NTR^ in microglia, as studies have suggested a subset of microglia express p75^NTR,^ and its expression is upregulated upon infection in mice [37]. Another study suggested no significant p75^NTR^ expression in microglia of wild-type mice, but the expression is increased in 5xFAD mice and Alzheimer’s patients [38], suggesting a potential role of BDNF-p75^NTR^ signaling in neuroinflammation. 

Cultured astrocytes have been shown to express BDNF, and the expression is increased by KCl, carbachol, and glutamate [39,40]. Astrocytic BDNF regulates neuronal dendrite complexity and dendritic spine number in vitro [17]. Studies have suggested that cortical layer II/III astrocytes can incorporate extracellular proBDNF through p75^NTR^-mediated endocytosis and convert proBDNF to mature BDNF [16]. But whether and what percentage of astrocytes produce BDNF in vivo under physiological conditions remain elusive. Our results show that a small subset of astrocytes express BDNF in the SS cortex and hippocampal CA1 under physiological conditions in mice. This is consistent with other studies that showed a relatively low baseline level of BDNF in astrocytes [41] but an upregulation of astrocytic BDNF expression under pathological conditions induced by MK-801, an N-methyl-D-aspartic acid (NMDA) receptor antagonist, in vitro [42], and treatment of glatiramer acetate in the R6/2 HD mouse model in vivo [41]. Although only 10.6% of hippocampal CA1 astrocytes express BDNF in adult mice under physiological conditions, astrocytes seem to be an important source of BDNF for normal CNS functions. Studies using BDNF knockdown mouse models found that astrocytic BDNF is required for oligodendrogenesis after white matter damage [43]. In the experimental autoimmune encephalomyelitis (EAE) model of multiple sclerosis, astrocyte-specific BDNF knockout led to more severe clinical course with increased axonal injury and loss [44]. 

It is known that astrocytes predominantly express TrkB.T, which plays important roles in mediating calcium release from intracellular stores in astrocytes [10] and controlling astrocyte morphological maturation [12,45]. In the adult hippocampal CA1 subregion, only less than 5% astrocytes express TrkB.FL, while more than 60% astrocytes express TrkB.T. These data are consistent with the current belief that astrocytes dominantly express TrkB.T in the adult mice. The role of astrocytic TrkB.T has been shown in various contexts. It has been reported that TrkB.T in astrocytes contributes to neuropathic pain following spinal cord injury [46] and regulates energy and glucose homeostasis in the ventromedial hypothalamus [14]. Reductions in astrocytic TrkB.T were suggested to be neuroprotective in a model of temporal lobe epilepsy [11]. Although only 3.6% of astrocytes express TrkB.FL in the adult CA1, we found that tracing with the *Ntrk2^2A-Cre^* allele from embryo to adult cumulatively labels 28.1% astrocytes in the region, suggesting that some astrocytes or their precursors transiently express TrkB.FL during development. This corroborates a previous in vitro study that showed radial glia (the main astrocytic precursor cells) and proliferating cortical astrocytes in culture express mRNAs for both TrkB.FL and TrkB.T, whereas differentiating astrocytes predominantly express TrkB.T and downregulate TrkB.FL expression [47]. However, our results indicate that only a small subset of astrocytic precursor cells express TrkB.FL, and, otherwise, all astrocytes will be marked by EGFP in *Ntrk2^2A-Cre/+^*;EGFP-L10a/+ mice. It would be intriguing to determine if astrocytes derived from this subset of precursor cells have any unique feature and what role TrkB.FL plays in the development of these astrocytes in future studies. 

Our data show that a very small subset (less than 5%) of oligodendrocytes in the hippocampal CA1 express BDNF. Contradictory to the studies that showed BDNF expression in cortical oligodendrocytes [48,49], our data showed that most Olig2^+^ cells in the cortex do not express BDNF. Oligodendroglial BDNF expression has been reported in a few studies. A study showed that oligodendrocytes in the spinal cord and optic nerve express *Bdnf* mRNA, and the expression is increased in Long-Evans shaker rats that lack CNS myelin due to an *Mbp* gene mutation [50]. Another study found that fingolimod treatment resulted in increased *Bdnf* mRNA concomitant with increased OPC differentiation in a rat OPC culture [51]. Our results confirm the expression of BDNF in oligodendrocytes in certain brain regions. And our observation of increased BDNF-expressing oligodendrocytes in AAV-injected mice may suggest that BDNF expression is increased when myelin is disrupted, as a previous study has reported increased BDNF levels in oligodendrocytes after injury [52]. 

Our results also showed that only OPCs, but not mature oligodendrocytes, express TrkB.FL and TrkB.T in the hippocampal CA1 of adult mice. BDNF-TrkB signaling in OPCs has been suggested to have important functions. OPC-specific TrkB deletion resulted in impaired activity-dependent myelination [19]. BDNF and its structural mimetic TDP6 have been shown to promote myelination and myelin repair via oligodendroglial TrkB receptor [21,53,54]. Future studies are necessary to determine which TrkB isoform(s) are responsible for mediating these effects. 

There are several limitations in this study. First, we only examined the glial expression of BDNF and TrkB in a few representative CNS regions, and our findings may not be applicable to all CNS regions. Second, this study was focused on the glial expression of BDNF and TrkB in the healthy CNS, which may be altered after injury or under pathological conditions. Finally, our genetic approach only determines whether a cell expresses a gene but cannot measure the expression level of the gene in the cell. 

Recent single-cell transcriptomic analyses reveal the heterogeneity of glial cells [55,56,57]. It is likely that the markers we used do not uncover all astrocytes and oligodendrocytes. Therefore, our analysis may underestimate the percentage of astrocytes or oligodendrocytes that express BDNF or TrkB. However, Iba1 is commonly used to identify transcriptomes of single microglial cells and should mark all known microglia. In summary, we characterized the expression of BDNF and TrkB in microglia, astrocytes, and oligodendrocytes in the current study. Our results clarify that microglia in the examined regions do not express BDNF or TrkB. Our findings also open opportunities to investigate previously unidentified roles of BDNF and TrkB in astrocytes and oligodendrocytes.

## Data Availability

Original data generated and analyzed during this study are included in this published article.
